# Effects of weight loss intervention on anxiety, depression and quality of life in women with severe obesity and polycystic ovary syndrome

**DOI:** 10.1038/s41598-024-63166-w

**Published:** 2024-06-12

**Authors:** Josefin Kataoka, Marie Olsson, Eva Lindgren, Ingrid Larsson, Johanna Schmidt, Anna Benrick, Elisabet Stener-Victorin

**Affiliations:** 1 Department of Physiology, Institute of Neuroscience and Physiology, Sahlgrenska Academy, University of Gothenburg, Box 430, 405 30 Gothenburg, Sweden; 2 Department of Obstetrics and Gynaecology, Sahlgrenska University Hospital, Gothenburg, Sweden; 3 Department of Physiology and Pharmacology, Karolinska Institute, Biomedicum, B5, 171 77 Stockholm, Sweden; 4 Department of Medicine, Sahlgrenska University Hospital, Gothenburg, Sweden; 5 Institute of Medicine, Sahlgrenska Academy, University of Gothenburg, Box 428, 405 30 Gothenburg, Sweden; 6 Department of Obstetrics and Gynaecology, Institute of Clinical Sciences, Sahlgrenska Academy, University of Gothenburg, 416 85 Gothenburg, Sweden; 7 Institute of Health Sciences, University of Skövde, Box 408, 541 28 Skövde, Sweden

**Keywords:** Polycystic ovary syndrome, Severe obesity, Health-related quality of life, Anxiety, Depression, Weight loss, Psychology, Human behaviour, Endocrine reproductive disorders

## Abstract

Polycystic ovary syndrome (PCOS) is a common endocrine disorder in women that is associated with an increased risk of anxiety and depression and with a lower health-related quality of life (HRQoL). PCOS is closely associated with obesity, which per se can lead to symptoms of anxiety and depression and lower HRQoL. The first-line treatment for PCOS is weight loss through lifestyle intervention, which has been shown to improve all symptoms of the syndrome. The aim of this study was to investigate symptoms of anxiety and depression and HRQoL in women with severe obesity (BMI ≥ 35) with and without PCOS, and to evaluate the effect of a one-year structured weight loss intervention. A total of 246 women with severe obesity (PCOS n = 63, non-PCOS n = 183) were included. The comprehensive psychopathological rating scale self-rating scale for affective symptoms (CPRS-S-A) and the short form-36 (SF-36) were used to assess symptoms of anxiety and depression and HRQoL. In total 72 women of the 246 women with severe obesity completed a one-year weight loss programme and were followed up and compared with baseline data. In women with severe obesity, there were no differences in symptoms of anxiety and depression and HRQoL between women with and without PCOS at baseline. Clinically relevant anxiety symptoms were present in 71.3% (PCOS) and 65.6% (non-PCOS), and depression symptoms were present in 56.4% (PCOS) and 52.2% (non-PCOS). Significant weight loss improved physical HRQoL in all women, but reduced symptoms of anxiety and depression only in women without PCOS. There were no differences when comparing the changes between the groups. Women with severe obesity are severely affected by symptoms of anxiety and depression, independent of PCOS. Weight loss improved symptoms of anxiety and depression in women without PCOS, but there were no differences between groups in change from baseline to follow-up**.**

**Trial registration number: **Clinical trial.gov: NCT01319162, March 18, 2011. Date of registration and enrolment of the first subject September 2011.

## Introduction

Polycystic ovary syndrome (PCOS) is a common endocrine disorder in women of reproductive age in which the main feature of the syndrome is hyperandrogenism. Clinical features associated with PCOS are hirsutism, oligo- or anovulation and polycystic ovarian morphology. Associated comorbidities with PCOS include infertility, type 2 diabetes and cardiovascular disease^1,2^. It is known that women with PCOS, regardless of BMI, have more symptoms of anxiety and depression and lower health-related quality of life (HRQoL) than women without the syndrome^3–8^. Overweight or obesity is present in the majority of women with PCOS, and exacerbates all symptoms of the syndrome^9^. According to genetic studies, obesity is considered causal for PCOS^10,11^. Obesity in itself is associated with anxiety and depression and lower HRQoL^12,13^, with a correlation found between increasing BMI and reduced HRQoL and symptoms of anxiety and depression^5,14,15^. The main treatment for PCOS is weight loss through lifestyle modification, which has been shown to improve all symptoms^15–17^. There are many studies on women with PCOS with BMI < 35, but there few studies on PCOS in women with severe obesity (BMI ≥ 35).

The primary aim of this study was to determine in women with severe obesity; whether women with PCOS have more symptoms of anxiety and depression and lower HRQoL than women without PCOS, and to examine the effect of a one-year structured weight loss intervention on HRQoL and symptoms of anxiety and depression. The second aim was to determine how symptoms of anxiety and depression and HRQoL differ across BMI categories, using data from previous studies conducted by the lab that included women with a wide range of BMI^18–21^.

## Participants and methods

Data from four studies conducted in Sweden have been used for this study^18–21^. All patients received oral and written information about the study and signed an informed consent allowing their data to be used in research.

The main study (Study 1)^18^, was a prospective clinical study, including women with severe obesity (BMI ≥ 35), referred for weight reduction to the obesity unit at Sahlgrenska University Hospital between 2011 and 2016. Participants in the main study were screened for PCOS using the National Institutes of Health (NIH)-criteria ^22^,were assessed with measurements of body composition, blood sampling for hormone analyses, and answered questionnaires. All women in the main study started a 12-month structured weight loss intervention including a very low-energy diet, as described below. After 12 months, baseline measurements were assessed in those who completed the intervention.

Three further studies (Study 2–4)^19–21^ included women in different BMI-categories, aged 18–38 years, recruited from the community between 2005 and 2013, where participants were screened for PCOS with the Rotterdam-criteria^23^.

### Data collection

Data was collected at baseline in Study 1–4, and at twelve months in Study 1.

#### Anthropometry and biochemistry

The studies collected body weight and height data, blood samples for analyses of testosterone (T) and sex hormone binding globulin (SHBG), retrospective self-reported menstrual data, and assessment of hirsutism with the Ferriman-Gallwey (FG)-score. Blood samples were analysed at the ISO-accredited laboratory (ISO 15,189:2012, ISO 22,870:2016) at Sahlgrenska University Hospital, Gothenburg, Sweden. Testosterone was measured with electrochemiluminiscent immunoassay with competitive analysis (ECLIA) (COBAS 8000 Roche Diagnostics Scandinavia AB, Sweden). The coefficient of variation (CV) was 6% at 2.0 nmol/L. The lower detection limit was 0.4 nmol/L. BMI was calculated using weight in kilos, divided with squared height in meters. Free testosterone (fT) was calculated using total testosterone and SHBG assuming a fixed albumin concentration of 4.3 g/liter^24^

#### Questionnaires

To assess symptoms of anxiety and depression the Comprehensive Psychopathological Rating Scale Self-rating Scale for affective symptoms (CPRS-S-A) was used. It is a well established questionnaire used in research and clinical practice for defining symptoms of anxiety and depression and to evaluate treatment^25^. The questionnaire contains 19 domains designed to measure symptoms of depression, anxiety, and obsessive–compulsive syndrome, where sensitivity to the latter is lower and therefore not used in this study. From CPRS-S-A, the subscales Brief Scale for Anxiety-self-rating (BSA-S) and Montgomery Åsberg Depression Rating Scale- self-rating (MADRS-S) are extracted and measure symptoms of anxiety (BSA-S) and depression (MADRS-S)^25,26^. These scales consist of nine domains each, of which two are present in both scales. All domains are rated on a six-point scale where 0 represents absence of symptoms, 2 represents a potentially pathological deviation, 4 represents a pathological condition, and 6 represents an extremely pathological condition. Domain ratings are summed to obtain a score with a maximum of 54 for each scale. To evaluate clinically relevant symptoms of anxiety and depression, a cut-off score ≥ 11 in each scale was used, a value which has been shown to discriminate health from disease^27–29^.

HRQoL is a quantitative score used to describe a person*’*s health status, including physical and mental health. Here we used the short form-36 (SF-36) which is a validated questionnaire, widely used in research and clinical practice^30^*.* It was developed to assess a person*’*s perception of how their health has affected their physical, mental, and social functioning over the past four weeks. The SF-36 is divided into eight domains and each domain is scored from 0 to 100*,* with 0 being the worst possible health quality, and 100 being the best possible health quality. Two summary scores are obtained:

the sum for the physical component (PCS) and the sum for the mental component (MCS) score, which we refer to as physical and mental HRQoL respectively.

### Intervention

The women in Study 1 began a 12-month structured weight loss intervention with a very low energy diet (VLED). The first 12 weeks consisted of a liquid diet of 450–800 kcal/day, followed by the reintroduction of solid foods according to a pre-calculated plan. During the intervention, all participants had regular meetings with nurses and dietitians who monitored wellbeing and weight and provided support and advice on dietary adherence. The details of the intervention have been described previously^18^. There was no planned psychological follow-up for the participants. However, all participants had an appointment with a doctor who could refer those who needed psychological help to appropriate care.

### Statistical analyses

SPSS 27.0 was used for statistical analyses. All data is presented as mean ± standard deviation (SD). The Mann–Whitney U-test was used to compare women with and without PCOS at baseline. Adjustment for age or BMI was calculated with ANCOVA. The Wilcoxon signed-rank test was used to analyze within-group changes from baseline to post intervention. In women with severe obesity, the Chi-2 test was used to compare rates of clinically relevant symptoms of anxiety and depression. Correlation analyses were made using Spearman’s rank correlation test. *P* < 0.05 was considered significant.

### Ethical approval and consent to participate

The included studies were performed in accordance with the Declaration of Helsinki. Ethical approval for the four studies included was received from the Ethics Committee, University of Gothenburg, Gothenburg, Sweden with the DNR-number 106-11, 679-08, 520-11 and 307-05, respectively. All participants gave their oral and written informed consent to participate in the study.

## Results

### Study 1: women with severe obesity

#### Baseline

In total, 246 women with severe obesity were included (PCOS n = 63, non-PCOS n = 183). Mean BMI was 39.9 ± 4.7 (PCOS) and 39.6 ± 4.7 (non-PCOS). Compared to women without PCOS, women with PCOS were younger (PCOS 33.0 ± 8.3 and non-PCOS 37.9 ± 8.2), and therefore all analyses were adjusted for age. Women with PCOS had higher circulating free testosterone and fasting insulin, more hirsutism as measured with a higher Ferriman-Gallwey score, and lower circulating SHBG^18^. There were no differences in symptoms of anxiety and depression between women with and without PCOS (Table 1). At baseline, clinically relevant anxiety symptoms (BSA-S score ≥ 11) were present in 71.3% of women with PCOS and 65.5% of women without PCOS, with no differences between groups (*p* = 0.256). Clinically relevant symptoms of depression (MADRS-S score ≥ 11) were present in 56.4% of women with PCOS and 52.2% of women without PCOS, with no differences between groups (*p* = 0.464). Moreover, there were no differences in the physical component (PCS) or the mental component (MCS) score of the SF-36 when comparing women with and without PCOS (Table 2).

**Table 1:** Symptoms of anxiety and depression in women with severe obesity (BMI ≥ 35) with and without PCOS at baseline**.**

	**PCOS n = 63**	**Non-PCOS n = 183**	***P*-value**	***P*-value adjusted for age**
BSA-S sum total	**17.5 ± 8.3**	**16.8 ± 9.3**	0.559	0.826
1. Feelings of unease	2.3 ± 1.5	2.1 ± 1.6	0.214	0.505
2. Irritability and anger	2.2 ± 1.6	2.1 ± 1.5	0.345	0.458
3. Sleep	2.5 ± 1.6	2.2 ± 1.6	0.209	0.126
4. Concern for health	1.7 ± 1.6	1.8 ± 1.7	0.750	0.359
5. Worry over trifles	2.1 ± 1.5	2.0 ± 1.8	0.823	0.732
6. Phobias	1.6 ± 1.6	1.6 ± 1.7	0.750	0.531
7. Physical discomfort	2.1 ± 1.6	2.0 ± 1.6	0.321	0.591
8. Aches and pains/Muscular tension	2.5 ± 1.5	2.7 ± 1.7	0.529	0.951
9. Panic attacks	0.5 ± 1.1	0.5 ± 1.1	0.637	0.737
MADRS-S sum total	**14.7 ± 8.9**	**13.8 ± 9.1**	0.531	0.597
1. Mood	1.3 ± 1.6	1.1 ± 1.3	0.645	0.386
2. Feelings of unease	2.3 ± 1.5	2.1 ± 1.6	0.214	0.505
3. Sleep	2.5 ± 1.6	2.2 ± 1.6	0.209	0.126
4. Appetite	0.7 ± 1.4	0.6 ± 1.2	0.933	0.764
5. Ability to concentrate	1.7 ± 1.4	1.7 ± 1.7	0.612	0.844
6. Initiative	1.7 ± 1.6	1.8 ± 1.7	0.765	0.843
7. Emotional involvement	1.2 ± 1.2	1.2 ± 1.2	0.649	0.812
8. Pessimism	2.5 ± 1.5	2.4 ± 1.6	0.622	0.560
9. Zest for life	0.8 ± 1.3	0.7 ± 1.2	0.877	0.986

Values are presented as means ± SD. P-value is calculated using Mann–Whitney U-test and *p*-value adjusted for age is calculated with ANCOVA. *P* < 0.05 was considered statistically significant. Statically significant *P*-values and summary scores on depression and anxiety are presented in bold.

Abbreviations: BMI, Body Mass Index; MADRS-S, Montgomery Åsberg Depression Rating Scale-self-rating; BSA-S, Brief Scale for Anxiety-self-rating.

Subscales BSA-S for anxiety and MADRS-S for depression, score range 0–54 where 0 represents the absence of symptoms.

**Table 2:** Health Related Quality of Life (HRQoL) was assessed with SF-36 in women with severe obesity (BMI ≥ 35) with and without PCOS at baseline.

	PCOS n = 63	Non-PCOS n = 183	*P*-value	*P*-value adjusted for age
Physical function	67.9 ± 20.6	65.6 ± 22.9	0.568	0.984
Role physical	59.5 ± 40.8	56.3 ± 38.4	0.594	0.868
Bodily pain	50.2 ± 22.7	51.6 ± 27.2	0.998	0.376
General health	43.1 ± 21.4	46.8 ± 22.4	0.301	0.124
Vitality	36.0 ± 22.6	34.4 ± 21.4	0.678	0.937
Social functioning	64.8 ± 26.7	61.7 ± 26.2	0.302	0.480
Role emotional	53.6 ± 40.3	59.4 ± 43.8	0.358	0.372
Mental health	56.1 ± 22.0	58.4 ± 21.1	0.576	0.520
*Physical component score (PCS)*	**40.3 ± 9.3**	**39.2 ± 11.1**	0.457	0.891
*Mental component score (MCS)*	**36.4 ± 14.2**	**37.7 ± 12.7**	0.537	0.646

Values are presented as means ± SD. P-value is calculated using Mann–Whitney U-test and p-value adjusted for age is calculated with ANCOVA. *P* < 0.05 was considered statistically significant. Summary domains are present in bold. Abbreviations: BMI, Body Mass Index; SF-36, Short form -36.

Score range 0–100 (low to high HRQoL).

#### After 12-months intervention

At 12 months, 73 women remained for follow-up (PCOS n = 16, non-PCOS n = 57). Comparing those who dropped out of the study with those who completed the study, there were no differences regarding symptoms of anxiety (*p* = 0.601) and depression (*p* = 0.590) or mental (*p* = 0.404) and physical (*p* = 0.600) quality of life. Women with and without PCOS lost weight (PCOS: 105.3 ± 13.8 kg to 92.9 ± 14.8 P = 0**.**001, non-PCOS 111.7 ± 15.3 kg to 98.2 ± 15.2 kg P < 0.001) and decreased in BMI (PCOS: 38.6 ± 2.3 to 33.7 ± 4.0 *p* = 0.001, non-PCOS: 39.6 ± 4.3 to 35.0 ± 5.4 *p* < 0.001). Mean weight loss after 12 months was approximately 12% in both groups. Women without PCOS had decreased free testosterone and increased SHBG, whereas women with PCOS did not change^18^. Women with PCOS improved in the emotional involvement domain of CPRS-S-A, but not in the total scores of symptoms of anxiety (BSA-S) and depression (MADRS-S) (Table 3**)**. Women without PCOS improved in both total scores of symptoms of anxiety (BSA-S), and total scores of symptoms of depression (MADRS-S) (Table 3**)**.

**Table 3:** Symptoms of anxiety and depression in women with severe obesity (BMI ≥ 35) with and without PCOS before and after weight reduction program.

	PCOS n = 16			non-PCOS n = 57			
	Before	After	*P*-value	Before	After	*P*-value	*P-* change
BSA-S sum total	**14.9 ± 8.4**	**14.4 ± 9.1**	0.887	**17.7 ± 9.6**	**13.7 ± 9.1**	** < 0.001**	0.095
1. Feelings of unease	2.4 ± 1.9	2.3 ± 2.0	0.753	2.2 ± 1.6	1.5 ± 1.5	**0.001**	
2. Irritability and anger	1.7 ± 1.3	1.9 ± 1.3	0.435	2.0 ± 1.5	1.8 ± 1.6	0.362	
3. Sleep	2.6 ± 1.4	2.0 ± 1.5	0.250	2.3 ± 1.5	2.0 ± 1.6	0.156	
4. Concern for health	1.2 ± 1.7	1.1 ± 1.1	0.809	2.0 ± 1.9	1.3 ± 1.4	**0.014**	
5. Worry over trifles	1.6 ± 1.7	1.8 ± 2.1	0.554	2.1 ± 1.8	1.6 ± 1.4	0.024	
6. Phobias	1.6 ± 2.0	1.2 ± 1.6	0.230	1.5 ± 1.6	1.2 ± 1.7	0.235	
7. Physical discomfort	1.6 ± 1.6	2.0 ± 1.8	0.167	2.2 ± 1.6	1.7 ± 1.8	**0.030**	
8. Aches and pains, muscular tension	2.1 ± 1.3	1.9 ± 1.7	0.721	2.9 ± 1.7	2.2 ± 1.9	**0.001**	
9. Panic attacks	0.3 ± 0.7	0.3 ± 0.9	0.785	0.5 ± 1.2	0.5 ± 1.1	0.776	
MADRS-S sum total	**12.9 ± 9.2**	**11.1 ± 8.3**	0.163	**15.0 ± 9.4**	**10.4 ± 8.2**	** < 0.001**	0.371
1. Mood	0.8 ± 0.9	0.5 ± 0.9	0.482	1.2 ± 1.4	0.7 ± 1.1	**0.003**	
2. Feelings of unease	2.4 ± 1.9	2.3 ± 2.0	0.753	2.2 ± 1.6	1.5 ± 1.5	**0.001**	
3. Sleep	2.6 ± 1.4	2.0 ± 1.5	0.250	2.3 ± 1.5	2.0 ± 1.6	0.156	
4. Appetite	0.5 ± 1.2	0.6 ± 1.3	0.492	0.8 ± 1.4	0.4 ± 0.8	**0.019**	
5. Ability to concentrate	1.3 ± 1.5	1.6 ± 1.9	0.399	1.9 ± 1.7	1.4 ± 1.4	**0.021**	
6. Initiative	1.7 ± 1.4	1.1 ± 1.0	0.067	1.9 ± 1.6	1.2 ± 1.4	**0.001**	
7. Emotional involvement	1.1 ± 1.2	0.6 ± 0.9	**0.013**	1.3 ± 1.3	1.0 ± 1.1	0.105	
8. Pessimism	2.0 ± 1.4	1.9 ± 1.9	0.690	2.6 ± 1.6	1.8 ± 1.5	**0.001**	
9. Zest for life	0.6 ± 1.1	0.5 ± 1.0	0.414	0.8 ± 1.2	0.5 ± 0.9	0.096	

Values are presented as means ± SD. P-value is calculated on values before and after weight reduction program in groups using Wilcoxon signed ranks test. *P* < 0.05 was considered statistically significant. *P*-change is comparing change (delta value) between groups and calculated using Mann–Whitney-U test. Statistically significant *P*-values and summary score on depression and anxiety are presented in bold.

Abbreviations: BMI, Body Mass Index; BSA-S, Brief Scale for Anxiety-self-rating, MADRS-S, Montgomery Åsberg Depression Rating Scale-self-rating.

Women with and without PCOS improved in the physical function domain of SF-36 and in the computed summary score for PCS, but not in the computed summary score for MCS (Table 4**)**.

**Table 4:** Health Related Quality of Life (HRQoL) was assessed with SF-36 in women with severe obesity (BMI ≥ 35) with and without PCOS before and after weight reduction program.

	PCOS n = 16			Non-PCOS n = 57			*P*-value change
	Before	After	*P*-value	Before	After	*P*-value	
Physical function	65.0 ± 23.4	84.7 ± 19.2	**0.001**	65.2 ± 21.7	76.0 ± 21.4	** < 0.001**	
Role physical	73.4 ± 34.7	70.3 ± 39.0	0.810	51.8 ± 39.2	68.9 ± 38.35	**0.012**	
Bodily pain	54.9 ± 24.5	66.3 ± 29.6	0.156	48.1 ± 28.2	61.9 ± 30.2	**0.001**	
General health	52.9 ± 16.5	64.4 ± 24.9	0.052	44.7 ± 22.7	59.5 ± 21.5	** < 0.001**	
Vitality	38.4 ± 24.7	45.9 ± 28.4	0.208	33.7 ± 18.3	48.5 ± 23.1	** < 0.001**	
Social functioning	77.3 ± 22.0	74.2 ± 26.0	0.569	58.6 ± 26.8	71.2 ± 24.3	**0.004**	
Role emotional	58.3 ± 41.3	52.1 ± 48.6	0.679	61.7 ± 37.5	70.2 ± 44.0	0.161	
Mental health	62.3 ± 18.6	63.5 ± 21.3	0.655	55.9 ± 20.1	66.6 ± 18.1	** < 0.001**	
*Physical component score (PCS)*	**41.5 ± 8.5**	**49.6 ± 8.8**	**0.011**	**38.7 ± 12.0**	**44.5 ± 10.9**	**0.001**	0.439
*Mental component score (MCS)*	**39.9 ± 13.9**	**38.1 ± 14.5**	0.605	**37.4 ± 12.2**	**41.3 ± 12.7**	0.074	0.215

Values are presented as means ± SD. P-value is calculated on values before and after weight reduction program in groups using Wilcoxon signed ranks test. *P* < 0.05 was considered statistically significant. *P*-change is comparing change (delta value) between groups and calculated using Mann–Whitney-U test. Statistically significant *P*-values and summary domains are presented in bold.

Abbreviations: BMI, Body Mass Index; SF-36, Short form-36.

Score range 0–100 (low to high HRQoL).

#### Study 1–4: women in a wide range of BMI-groups

Using the combined data (n = 407) from women with severe obesity (Study 1), with three earlier studies (Study 2–4)^19–21^, HRQoL and symptoms of anxiety and depression were determined in women with and without PCOS (PCOS n = 179, non-PCOS n = 228) with BMI ranging from 18.2 to 58.7 kg/m^2^.

**Figure 1:**
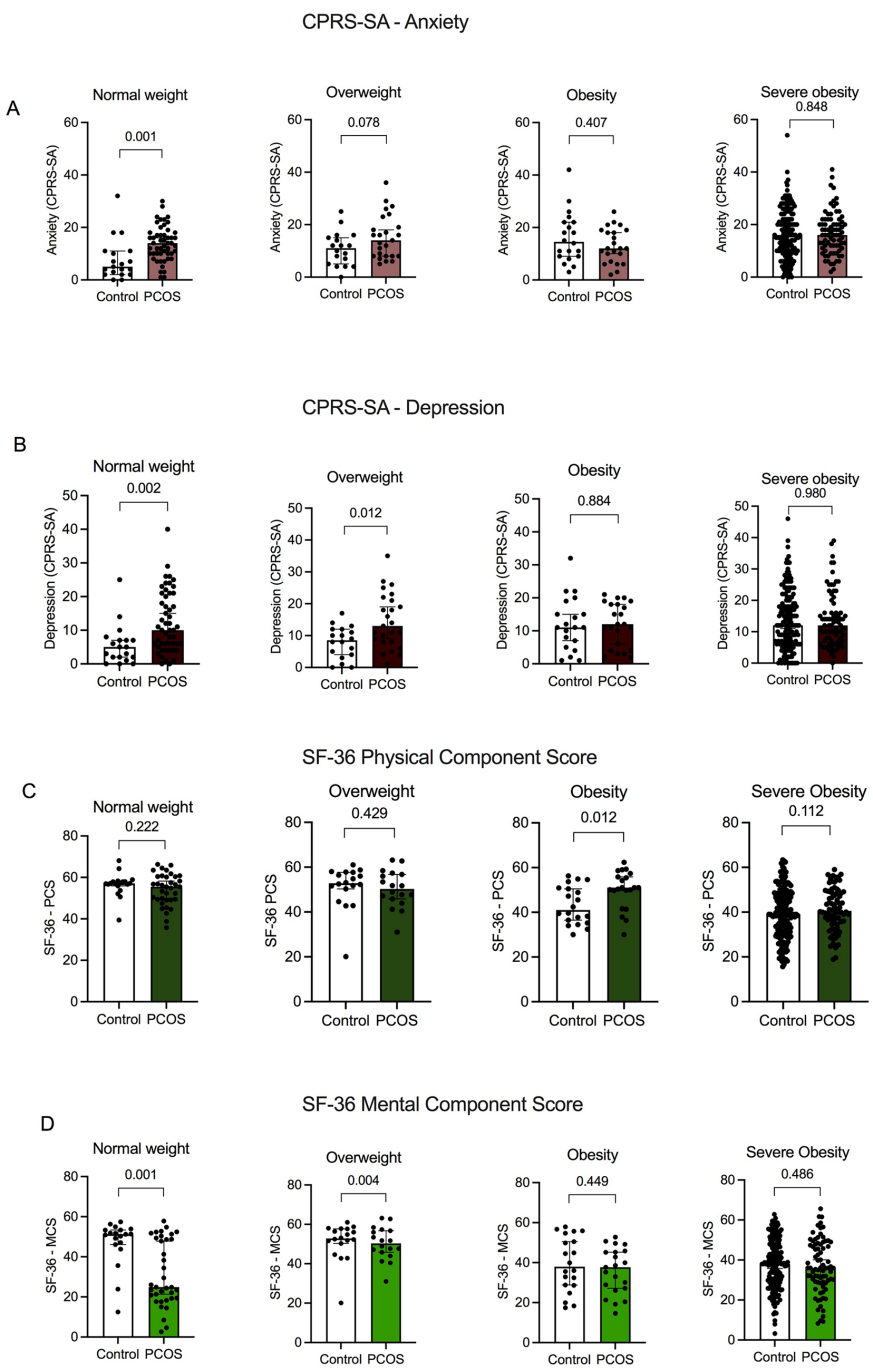
**A-D** Symptoms of (**A**) anxiety and (**B**) depression and (**C**) physical and (**D**) mental HRQoL in women with and without PCOS in different weight categories. Values are presented as mean ± SD. *P*-value is calculated using Mann–Whitney U-test and *p*-value adjusted for age and BMI is calculated with ANCOVA. *P* < 0.05 was considered statistically significant.

Women with PCOS with normal weight and overweight had more symptoms of depression and lower MCS compared to women without PCOS, and women with normal weight and PCOS had more symptoms of anxiety compared to women without PCOS (Fig. 1A-C). In women with obesity and severe obesity, there were no differences between groups regarding symptoms of anxiety and depression, or MSC (Fig. 1A-B**)**. Of note, in women with obesity, those without PCOS had a lower PCS **(**Fig. 1D**)**.

Correlation analyses were performed including all women, independent of PCOS diagnosis, with BMI < 30 and BMI ≥ 30 separated. In women with BMI < 30, FG-score was positively associated with symptoms of anxiety (r_s_ = 0.366, *p* < 0.01) and depression (r_s_ = 0.326, *p* < 0.01), and negatively associated with PCS (r_s_ = − 0.207, *p* < 0.05) and MCS (r_s_ = − 0.452, *p* < 0.01). Insulin was positively correlated to anxiety (r_s_ =0.222, p<0.05) and depression (r = 0.220, *p* < 0.05) and negatively correlated with PCS (r_s_ =-0.326, *p* < 0.01).

In women with BMI ≥ 30 there was a negative association between symptoms of anxiety and depression and free testosterone (r_s_ = − 0.131 and r_s_ = -0.144, *p* < 0.05), a positive correlation between symptoms of anxiety and depression and insulin (r_s_ = 0.127 and r_s_ = 0.116, *p* < 0.05), and a negative correlation between FG score and MCS (r_s_ = − 0.124, *p* < 0.05).

In women with PCOS and BMI < 30 free testosterone was positively correlated to MCS (r_s_ = 0.282, *p* < 0.01, and insulin was negatively correlated to PCS (− 0.311, *p* < 0.05).

## Discussion

Women with PCOS report more symptoms of anxiety and depression and a lower HRQoL than women without PCOS. Previous studies have found an association between increasing BMI and a lower HRQoL and more symptoms of anxiety and depression in women with PCOS^5,14,31^.

We found that women with PCOS with had more symptoms of anxiety and depression compared to women without PCOS, only for those with normal weight and overweight, with no differences for women with obesity or severe obesity. The lack of difference in the latter BMI groups is likely due to the fact that obesity per se affects HRQoL and symptoms of anxiety and depression. The results from this part of the study confirm and extend the findings of previous studies indicating symptoms of anxiety and depression and lower HRQoL are more common in normal-weight and overweight women with PCOS than in women without PCOS^3–6^. This contrasts with a meta-analysis in which women with PCOS had more symptoms of anxiety and depression and lower HRQoL than controls after adjustment for BMI^5^. The finding is however consistent with a recent study in women with severe obesity, in which women with and without PCOS did not differ in HRQoL^32^.

In all women with normal weight and overweight, independent of PCOS-diagnosis, FG-score was associated with symptoms of anxiety and depression, whereas in all women with obesity and severe obesity, free testosterone was negatively associated with symptoms of anxiety and depression. Similar findings have been reported, where more depressive symptoms in women with PCOS correlated to lower levels of fT^33,34^. Further, insulin was positively correlated to symptoms of anxiety and depression regardless of BMI. Previous studies have reported that neuropsychiatric issues such as anxiety and depressive behavior are common factors in patients with metabolic disease^35^, and that insulin is correlated to depressive symptoms^36^. This has also been shown in women with PCOS^37^, but the exact mechanism is not fully understood. The amygdala is a region in the brain that regulates emotional memory, anxiety, and cognitive function^38^. Since insulin was positively associated with symptoms of anxiety and depression, we hypothesize that insulin resistance can have effects in the amygdala. Moreover, brain insulin resistance has been shown to alter dopamine turnover and induce anxiety- and depressive-like behaviors in mice^39^. Insulin-sensitizing medications have been used in psychiatric practice to treat weight gain and other metabolic side effects that accompany the use of some antidepressant SSRI drugs^40^. Interestingly, recent studies show that metformin and glitazones can cause improvement in mental health^40–42^.

An important finding of this study is that women with severe obesity are strongly affected by symptoms of anxiety and depression. In women with severe obesity, regardless of PCOS diagnosis, the mean anxiety score was 17–18, and mean depression score was 14–15, which is higher than the mean score in the general population. In the general population, mean anxiety score is around 4 and the mean depression score is around 3^27,29^.

The majority of women with severe obesity in the present study had clinically relevant symptoms of anxiety and depression symptoms with a score in BSA-S or MADRS-S of 11 or more^27,29^. Women with severe obesity, regardless of PCOS diagnosis, had mean physical and mental HRQoL scores in the range of 35–40, which is lower than those in the general Swedish female population, who have mean scores of around 50^46^. These results are consistent with previous findings showing that obesity per se is associated with symptoms of anxiety and depression and impaired HRQoL^12,13^.

The structured weight loss intervention resulted in an average weight loss of about 12% in women with severe obesity both with and without PCOS^18^, with no difference between groups. This is a larger weight loss than the 5–10% in previous studies that have been shown to alleviate the symptoms of PCOS^47^. In women with PCOS and severe obesity, there were no improvements in symptoms of anxiety and depression after the intervention, which contradicts the results of previous studies^14,48^. Of note, symptoms of anxiety and depression improved only in women without PCOS. All women improved in physical but not mental HRQoL, although women without PCOS improved in several domains. These findings are in contrast to previous studies on women with obesity and PCOS, in which weight loss led to improvement in several domains of the SF-36^15,49^. However, a more recent study of HRQoL in women with severe obesity showed no difference in improvement between women with and without PCOS after weight loss^50^.

The mechanisms of psychiatric symptoms in women with severe obesity without PCOS appear to be strongly related to obesity, so that weight loss is more favorable for these women. It is possible that in women with PCOS and severe obesity, a weight loss of about 12% is not enough to see an impact on mental HRQoL and symptoms of anxiety and depression. Another possibility is that multiple factors contribute to symptoms of anxiety and depression and lower HRQoL in women with PCOS and that additional treatment is needed to alleviate these symptoms and improve mental HRQoL. A recent randomized controlled trial investigating cognitive behavioural therapy in women with PCOS, showed both a reduction in symptoms of anxiety and depression and an improvement in HRQoL after treatment^51^. One important explanation why no improvements were observed in the women PCOS is the high proportion of dropouts in the women with PCOS.

Indeed, a limitation of this study is the high drop-out rate from the weight reduction program. Of the 246 women who started the program, only 73 remained until the follow-up examination. Therefore, caution is needed when interpreting the results of the intervention. The lack of improvement in women with PCOS may be due to the fact that only 16 women remained in this group at follow-up. Although the dropout rate was high compared to previous weight loss studies^52^, the dropouts did not differ in symptoms of anxiety and depression and HRQoL from those who adhered to the intervention. A further limitation is that women with severe obesity were recruited from an obesity center referred for weight loss support and could therefore be more affected by their obesity in terms of comorbidities. One particular strength of this study over previous work is the inclusion of women with PCOS in BMI-categories ranging from normal weight to severe obesity.

As recommended by the International PCOS guideline and Androgen Excess and PCOS Society in 2023, all women with PCOS should be screened for anxiety and depression^53^. The recommended treatment for women with severe obesity and PCOS is lifestyle changes leading to sustainable weight loss to improve physical HRQoL. However, a greater weight loss than the recommended 5–10%^47,54^ may be needed for women with PCOS and severe obesity to alleviate symptoms of anxiety and depression and to improve their mental HRQoL. Additional treatment for these symptoms should be considered.

## Conclusions

In women with severe obesity, symptoms of anxiety, depression and HRQoL do not differ between women with and without PCOS. It is noteworthy that a high proportion of women with severe obesity suffer from clinically relevant anxiety and depression symptoms regardless of PCOS-status. After significant and similar weight loss in both groups, only women without PCOS improved in symptoms of anxiety and depression.

## Data Availability

The datasets used and/or analysed during the current study are available from the corresponding author upon reasonable request.

## Abbreviations

PCOS: Polycystic ovary syndrome
HRQoL: Health related quality of life
BMI: Body mass index
NIH: National institutes of health
T: Testosterone
SHBG: Sex hormone binding globulin
FG-score: Ferriman–Gallwey-score
ECLIA: Electrochemiluminiscent immunoassay with competitive analysis
CPRS-S-A: Comprehensive Psychopathological Rating Scale (CPRS) Self-rating Scale for affective symptoms
fT: Free testosterone
BSA-S: Brief scale for anxiet-self-rating
MADRS-S: Montgomery Åsberg Depression Rating Scale-self-rating
PCS: Physical component summary score
MCS: Mental component summary score
VLED: Very low energy diet
SPSS: Statistical package for the social sciences
SD: Standard deviation
ANCOVA: Analysis of co-variance
